# Supplementary material for: Luminescent κ-Carrageenan-Based Electrolytes Containing Neodymium Triflate
Source: Molecules. 2019 Mar 14;24(6):1020. doi: 10.3390/molecules24061020 (PMC6471547; doi:10.3390/molecules24061020)
Supplement: Supplementary file 1 [file molecules-24-01020-s001.pdf]

molecules

Luminescent κ-Carrageenan-based electrolytes containing neodymium triflate

S. C. Nunes [1,2,*], S. M. Saraiva [1], R. F. P. Pereira [3], M. M. Silva [3], L. D. Carlos [4], P. Almeida [2,5], M. C. Gonçalves [1,6], R. A. S. Ferreira [4] and V. de Zea Bermudez [1,6,*]

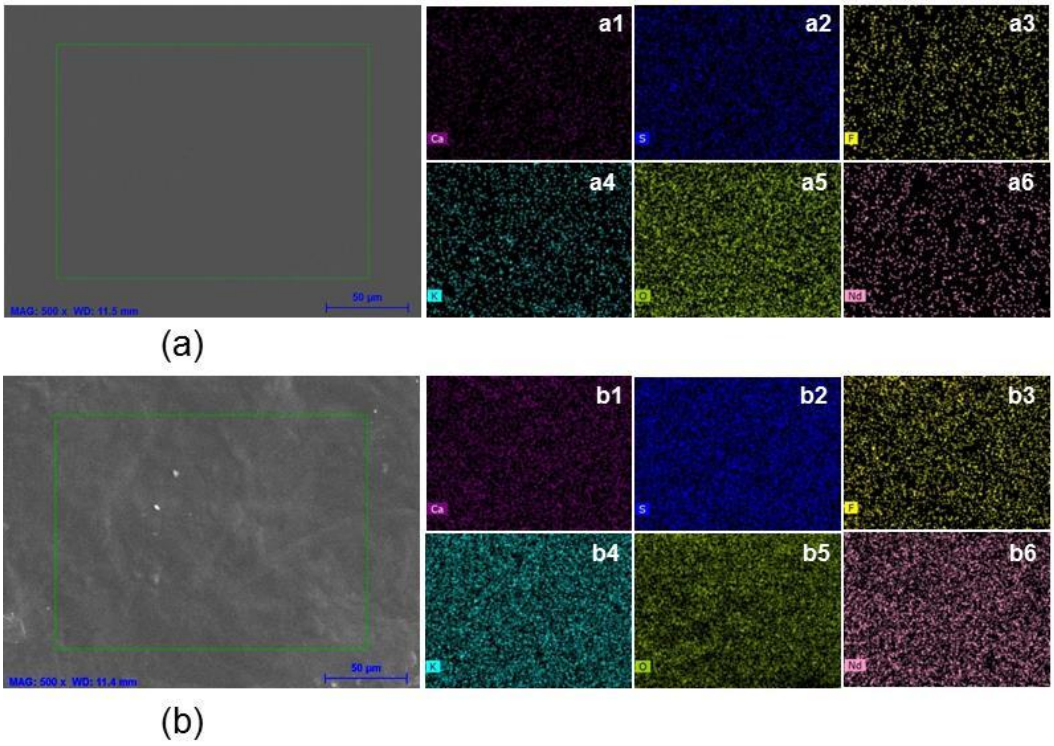

Figure S1: SEM images of selected CG$_{50}$Nd$_z$ membranes with z = 20 and 40 % (b). EDS mapping images for z = 20 % (a1-a6) and z = 40 % (b1-b6) for Ca (calcium, purple), S (sulphur, blue), F (fluor, yellow), K (potassium, cyanide), O (oxygen, green), and Nd (neodymium, pink) atoms.

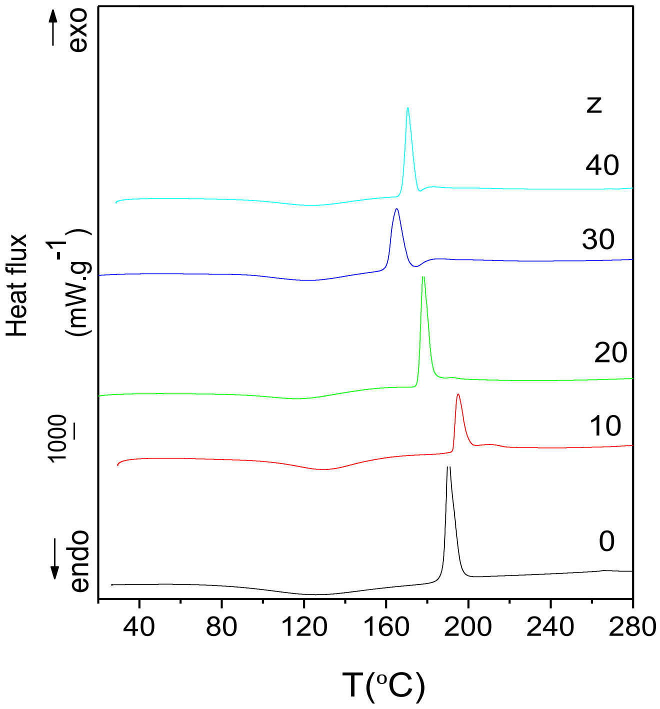

Figure S2: DSC curves of selected $CG_{50}Nd_z$ membranes
